# Effects of Long-Term Fertilization Regimes on Crop Yield Stability and Grain Quality in Maize and Winter Wheat Rotation of Northern China

**DOI:** 10.3390/plants15132018

**Published:** 2026-06-30

**Authors:** Wenjuan Cheng, Wei Gao, Mingyue Li, Hui Xiao, Juan Li, Qiang Zhang

**Affiliations:** 1 College of Resource and Environment, Shanxi Agricultural University, Taiyuan 030801, China; 13116119063@163.com; 2 Soil Health Laboratory in Shanxi Province, Institute of Eco-Environment and Industrial Technology, Shanxi Agricultural University, Taiyuan 030801, China; 3 Institute of Agricultural Resources and Environment, Tianjin Academy of Agricultural Sciences, Tianjin 300384, China; limingyuetj@163.com (M.L.); xiaohui-81@163.com (H.X.); 4 State Key Laboratory of Efficient Utilization of Arable Land in China, Key Laboratory of Plant Nutrition and Fertilizer, Ministry of Agriculture and Rural Affairs, Institute of Agricultural Resources and Regional Planning, Chinese Academy of Agricultural Sciences, Beijing 100081, China

**Keywords:** long-term fertilization, crop yield, yield variability, grain quality, crop rotation

## Abstract

The long-term effects of organic and inorganic fertilizer application on crop yield stability and grain quality were investigated in a maize–winter wheat rotation system in Tianjin, northern China, based on a continuous field experiment initiated in 1979. Forty-five years of data (1979–2023) were analyzed across six fertilizer treatments: an unfertilized control (CK); nitrogen only (N); nitrogen + phosphorus (NP); nitrogen + phosphorus + potassium (NPK); farmyard manure only (M); and nitrogen combined with high-rate manure (NM). The results indicated that the NM treatment yielded the highest crop productivity for both maize and winter wheat, with grain yields increasing by 81.6% and 162.6%, respectively, relative to the N treatment. Manure application significantly improved yield stability: the coefficient of variation (CV) of the winter wheat grain yield was the lowest under the M treatment, whereas maize grain yield exhibited the highest stability under the NM treatment. Grain quality analyses revealed that the N treatment significantly increased the wet gluten and protein content in winter wheat grain by 40.43% and 13.6%, respectively, relative to CK; the sedimentation value followed a similar trend. However, starch content remained statistically unchanged across all treatments. Collectively, the long-term combined application of nitrogen fertilizer and manure can steadily increase crop yield, mitigate inter-annual yield variability, and have no adverse effects on grain quality. These findings indicate that integrated N + manure fertilization is a more robust and sustainable alternative to sole chemical or sole organic fertilization for achieving high, stable yields and maintaining grain quality in intensive cereal production systems.

## 1. Introduction

In modern agricultural systems, fertilization plays a critical role in enhancing both the magnitude and temporal stability of crop yields. Currently, fertilizers are broadly categorized into two major classes: synthetic (mineral) fertilizers and organic fertilizers. Extensive research has demonstrated that synthetic fertilizers—particularly those supplying nitrogen (N), phosphorus (P), and potassium (K)—efficiently meet essential nutrient demands for crop growth and consistently increase grain yields [1,2,3]. For instance, Janna et al. [4] identified mineral N fertilizer application as the dominant driver of interannual yield stability in winter wheat, while Ma et al. [5] reported substantial maize yield gains in northeastern China through optimized mineral nutrient management. Nevertheless, prolonged reliance on synthetic fertilizers has been associated with adverse effects on soil physical structure, chemical buffering capacity, and biological health—including reductions in soil microbial biomass and functional diversity [6,7]. In response, organic fertilizers, especially livestock manure, have gained increasing adoption as complementary or alternative nutrient sources [8]. Empirical evidence indicates that long-term manure application enhances soil organic carbon content, improves aggregate stability, and increases plant-available nutrient pools [9,10]. Moreover, such practices have been shown to boost the yields of multiple crops: lucerne forage biomass increased significantly under multi-year manure regimes [11], and winter wheat grain yield rose markedly following sustained organic fertilizer inputs [12]. However, yield responses to organic fertilization are not universally positive; several field studies have documented yield declines relative to synthetic fertilizer controls [13,14]. This variability in agronomic performance is likely attributable to contextual factors—including feedstock composition and the maturity of organic amendments, prior soil fertility status, climatic water availability, and inherent soil properties [15,16,17]. Most comparative studies of organic and mineral fertilization rely on short-term field trials [14,18]. Long-term experimental datasets spanning over a decade are rarely reported [19], which limits mechanistic insights into the dynamic shifts in crop productivity and yield stability under sustained nutrient input strategies. Accordingly, the yield gap between organic and mineral systems may gradually narrow over time due to cumulative soil fertility improvements and adaptive edaphic processes.

Consequently, the observed yield gaps between organic and inorganic systems may narrow over time, reflecting adaptive soil processes and cumulative fertility effects. Furthermore, the long-term synergistic potential of integrated organic–inorganic fertilization remains underexplored. For example, Ahrends et al. [20] demonstrated that severe potassium (K) deficiency and complete fertilizer omission drastically reduced winter wheat yield stability, whereas balanced NPKCa fertilization markedly enhanced it. While optimized synthetic fertilization alone can sustain yield stability, integrating it with organic inputs—such as manure or compost—further amplifies both absolute yield and its interannual consistency by improving soil nutrient supply capacity and buffering against environmental stress [21]. Consistent with this, Chen et al. [22] reported significantly higher yield stability for winter wheat under combined organic–inorganic application compared with either sole organic or sole NPK treatments. Collectively, these findings underscore that the fertilization strategy is a pivotal determinant of both productivity and resilience in cereal systems.

Wheat is a globally vital staple crop and ranks third in both production and harvested area among China’s major cereals—following rice and maize [23]. As consumer demand increasingly emphasizes nutritional quality, sensory attributes (e.g., taste and texture), and health-promoting properties, grain quality parameters—particularly those governing end-use functionality such as baking and processing performance—have become critical evaluation criteria. A substantial body of evidence indicates that fertilizer management significantly modulates wheat grain quality: long-term N fertilization consistently enhances protein concentration and gluten strength [24,25], thereby improving dough elasticity and bread-making quality. In contrast, Mäder et al. [26] reported no statistically significant differences in key nutritional indices (e.g., protein content, amino acid profile) or functional baking traits (e.g., loaf volume, crumb structure) between organically and conventionally grown wheat—suggesting that fertilizer sources alone do not deterministically govern final grain quality. In conclusion, these findings highlight that the effects of long-term fertilization on wheat are multifaceted, influencing not only yield magnitude and interannual stability but also compositional and functional quality attributes. Clarifying these integrated responses is therefore essential for developing nutrient management strategies that simultaneously ensure food security, nutritional adequacy, and market-relevant processing performance.

This study aimed to (1) quantify the long-term effects of organic versus inorganic fertilizer management on both grain yield and interannual yield stability in a winter wheat–maize rotation system in the North China Plain (Tianjin City), and (2) assess how these contrasting fertilization regimes influence key wheat grain quality attributes—including protein concentration, wet gluten content, sedimentation value, and processing-relevant functional properties. The optimization of fertilizer management strategies based on these findings offers a robust scientific foundation for enhancing both agronomic efficiency and grain quality in cereal production systems.

## 2. Results

### 2.1. Yield Performance of Various Fertilization Treatments

Based on the different organic fertilizer types used, the experiment was divided into three stages. We analyzed wheat and maize yields for each treatment during the periods of 1979–1998 (soil-amended farmyard manure), 1998–2015 (chicken manure), and 2015–2023 (certified commercial organic fertilizer) (Table 1).

In the first stage (1979–1998), the yields of wheat and maize were the highest under the NPK and NM treatments. The wheat yield ranged from 4852.0 to 5688.4 kg ha^−1^ and the maize yield ranged from 5018.4 to 6591.2 kg ha^−1^. The NPK treatment produced slightly higher yields than the NM treatment, but the difference was not significant. The CK produced the lowest yields: the wheat yield was 1376.0 to 2205.6 kg ha^−1^ and the maize yield was 1608.6 to 2984.4 kg ha^−1^, which were significantly lower than those of all other fertilization treatments. The results indicated that fertilization exhibited a prominent yield-promoting effect. The yield under sole N application was relatively low, being only slightly higher than that of the control treatment (CK).

In the second stage (1999–2015), the wheat and maize yields under the NM treatment were 4479.8 to 5598.8 kg ha^−1^ and 6271.0 to 10,758.4 kg ha^−1^, respectively. For the first time, NM outperformed NPK and yielded significantly more than the other treatments, demonstrating the advantages of the combined application of organic and inorganic fertilizers. The yield of the sole N treatment decreased markedly and showed no significant difference compared with the control treatment (CK).

In the third stage (2016–2023), the superiority of the NM treatment was further enhanced, with its yields being significantly higher than those of all other treatments. The yield under sole N application remained at a low level throughout this period.

### 2.2. Yield Stability Analysis

**Figure 1:**
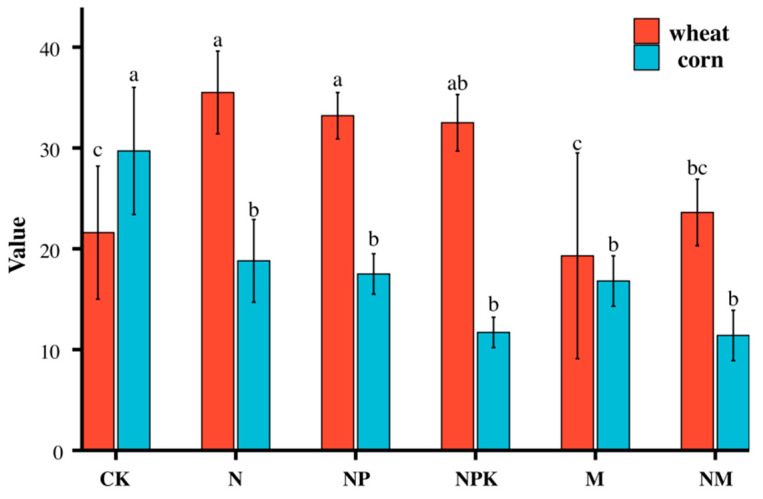
Yield variability in different treatments of winter wheat and corn. Note: CK (no fertilization), N (nitrogen), NP (nitrogen and phosphorus), NPK (nitrogen, phosphorus, and potassium), M (manure), NM (nitrogen plus manure combination). The error bar represents the standard deviation of the mean yield of the same treatment, and the different letters on the bar chart represent significant differences in the level (*p* < 0.05).

Figure 1 presents the effects of long-term fertilizer application on crop yield stability. Relative to CK, both organic and inorganic fertilizer applications significantly enhanced maize yield stability (*p* < 0.05). The coefficient of variation (CV) for maize yield across treatments followed the order: NM < NPK < M < NP < N, with the NM treatment exhibiting the lowest CV (12.04%). In contrast, for winter wheat, the M and NM treatments were associated with a marginally higher yield risk than CK; however, all chemically fertilized treatments—including N, NP, NPK, and NM—collectively conferred a significantly greater yield risk relative to CK. Notably, nitrogen-only fertilization (N) resulted in a comparatively high yield risk for both crops (winter wheat: 35.94%; maize: 30.25%) alongside low overall system productivity in the maize–winter wheat rotation. Among all treatments, the NM regime yielded the lowest integrated yield risk for the rotation system, whereas the highest maize-specific yield risk occurred under CK and the highest winter wheat-specific yield risk under the N treatment.

### 2.3. Core Quality Traits of Winter Wheat Grain

**Figure 2:**
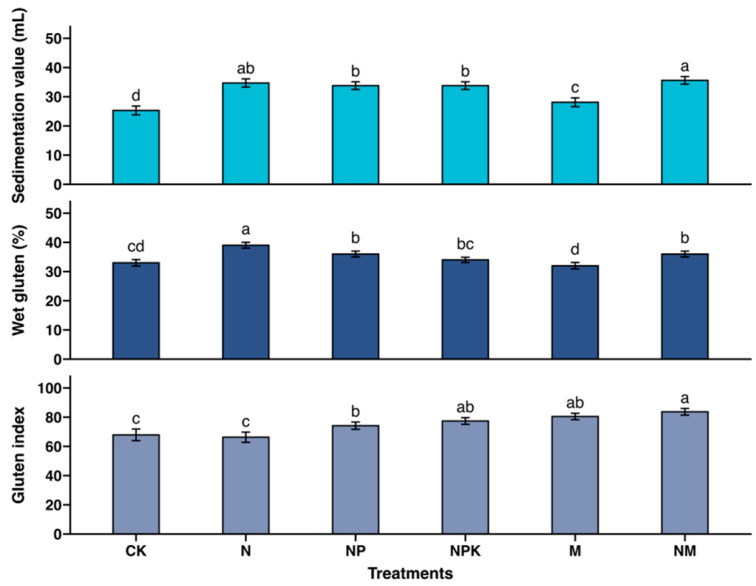
Grain qualities in different treatments of winter wheat. CK (no fertilization), N (nitrogen), NP (nitrogen + phosphorus), NPK (nitrogen + phosphorus + potassium), M (manure), NM (nitrogen and manure combination). Different letters at the same index represent significant differences under different treatments (*p* < 0.05).

Figure 2 illustrates the effects of long-term fertilization treatments on winter wheat grain quality parameters in 2020. Relative to CK (34.63%), the wet gluten content increased by 5.80%, 2.57%, 1.20%, and 2.47% under the N, NP, NPK, and NM treatments, respectively; the M treatment yielded the lowest value (33.13%), whereas the N treatment produced the highest (40.43%). The sedimentation volume was greatest under the NM treatment, followed sequentially by N, NPK, NP, and M—all significantly exceeding the CK value (*p* < 0.05). The gluten index was significantly enhanced by the NM, M, NPK, and NP treatments, with increments of 16.00, 13.66, 9.66, and 7.66 percentage points, respectively; in contrast, no significant difference was observed between the N and CK. The protein content was significantly elevated under the inorganic fertilizer treatments (N, NP, NPK) and the NM treatment relative to CK (Table 2), reaching a maximum of 13.6% under N; conversely, the M treatment reduced protein content by 0.3% relative to CK. Starch content showed no statistically significant variation across treatments (*p* > 0.05).

## 3. Discussion

### 3.1. Effects of Long-Term Fertilizer Treatments on Crop Yield

The combined application of nitrogen and manure (NM) significantly increased the winter wheat yield (5048 kg ha^−1^) and maize yield (7313 kg ha^−1^) relative to all other treatments. This finding aligns with Wei et al. [27], who reported substantially greater yield responses in both wheat and maize to integrated organic–inorganic fertilization than to sole applications of either organic or mineral fertilizers. Although the NPK treatment yielded less than NM, it outperformed both the N and NP treatments for both crops. Consistent with Niu et al. [28] and He et al. [29], potassium (K) supplementation significantly enhanced the maize yield compared with the K-deficient controls—indicating that an adequate exogenous K supply is critical for sustaining K nutrition during maize growth and maximizing grain production. Supporting this, Vyn et al. [30] observed increased maize grain yields under K fertilization in zero-tillage and mulch-tillage systems in Canada. A comparable K-mediated yield enhancement was also evident in winter wheat under the NP and NPK treatments. In contrast, the N-only treatment produced lower yields than anticipated—contrary to the expectation that nitrogen alone might sustain moderate productivity. This attenuated response is likely attributable to a suboptimal soil fertility status, particularly the low indigenous K and phosphorus availability, rendering the N-only input insufficient to support high-yielding crop performance—conditions closely resembling those in the control (CK) treatment.

Manure application enhances soil fertility and crop growth conditions, thereby contributing to increased grain yield [31]. In maize–winter wheat rotation systems, an elevated nitrogen demand is required to sustain both crop growth and high-yielding performance [32]. Notably, exceptionally low yields of both winter wheat and maize were observed across all fertilization treatments during 1994–1998 (Figure 2), which was likely attributable to adverse climatic conditions—including below-average temperatures and erratic precipitation patterns—during that period [33]. Fertilization remains a fundamental agronomic practice for securing stable and productive crop yields [1,2,3]. However, fertilizer management alone is insufficient; abiotic environmental factors—including temperature, solar radiation, and precipitation—exert substantial and often interactive effects on yield formation. Consequently, optimizing the synergistic balance among nutrient supply, crop physiology, and prevailing environmental conditions is essential for sustaining high and stable productivity in cereal rotations such as maize–winter wheat.

### 3.2. Effects of Long-Term Fertilizer Treatments on Yield Stability

Yield stability in the maize–winter wheat rotation system was evaluated across the fertilizer treatments, with treatment effects rigorously assessed using the CV as a quantitative stability metric. Maize yield exhibited high stability under the NM (CV = 12.04%), NPK (CV = 12.26%), and M (CV = 17.21%) treatments, whereas winter wheat yield stability was greatest under M (CV = 19.77%), CK (CV = 21.94%), and NM (CV = 24.06%). Collectively, these results indicate that organic–inorganic integration—particularly the NM and M treatments—reduced the overall yield risk in the rotation system. Consistent with Janna et al. [32], the mineral nitrogen (N) supply emerged as a principal determinant of winter wheat yield stability. Likewise, Chloupek et al. [34] demonstrated that optimizing plant-available N—regardless of the source (inorganic, manure-derived, or rotation-induced mineralization)—enhances yield stability across diverse cropping systems. Furthermore, Berzsenyi et al. [35] reported improved yield stability under combined mineral and manure fertilization, a finding corroborated by the superior performance of the NM treatment in the present study.

**Figure 3:**
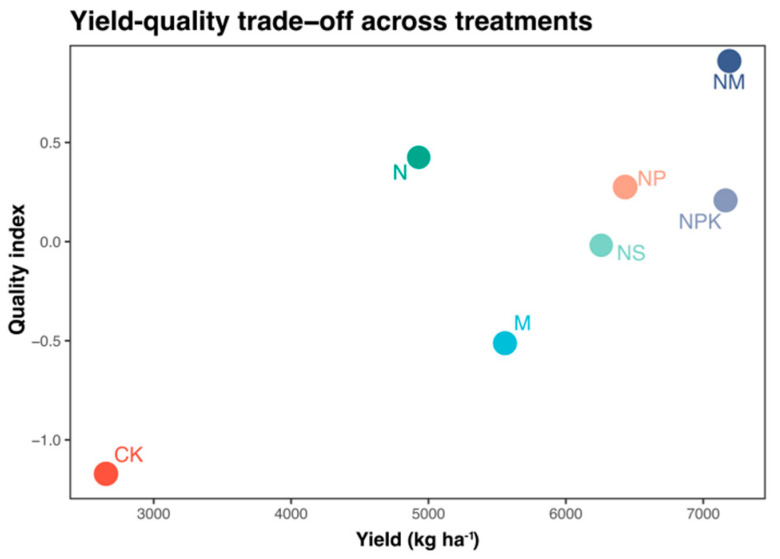
The trade-off analysis between long-term average maize yield and grain quality index under different fertilization regimes. Note: The x-axis represents the mean maize yield averaged over 45 years (1979–2023). The y-axis denotes the composite quality index, which was calculated based on three key parameters: wet gluten content, gluten index, and sedimentation value. To ensure comparability, the raw data of these three parameters were standardized using Z-scores and then summed with equal weighting (1:1:1). Different colors and letters represent specific fertilization treatments.

In northern China, winter precipitation is typically insufficient to meet crop water requirements during the overwintering growth stage (Figure 1), and low soil temperatures further impede root physiological activity—thereby limiting early-season growth resumption. Consequently, the combined stress of cold temperatures and relative soil moisture deficit likely contributed to interannual yield instability under uniform fertilization regimes. Effective nutrient management remains essential for sustaining both high and stable crop yields [33]. Notably, the manure-only (M) treatment consistently outperformed the mineral-fertilizer-only treatments across the entire experimental period; this advantage was particularly pronounced in 2018, when most other treatments exhibited marked yield declines (Figure 2). Organic fertilizer application enhances multiple soil functions: it improves soil structure [36], increases water-holding capacity [37], and elevates the soil organic matter content [10]—collectively buffering crop performance against climatic variability. Consistent with this mechanistic understanding, the M treatment demonstrated superior yield stability in the maize–winter wheat rotation system (Figure 3). Furthermore, Manns and Martin [38] reported that favorable edaphic and nutritional conditions enhance crop resilience to abiotic environmental stressors.

### 3.3. Effects of Long-Term Fertilizer Treatments on Winter Wheat Qualities

Crop quality has emerged as a key agronomic and nutritional priority, yet it remains highly sensitive to multiple interacting factors—including ecological conditions, fertilization regimes, and cultivar genotype [39]. Strategic fertilizer management constitutes a critical lever for modulating grain quality attributes, particularly those relevant to end-use functionality (e.g., milling, baking) and nutritional composition—most notably protein concentration and gluten quality [40]. Starch represents the predominant storage carbohydrate in wheat grain, accounting for approximately 70–75% of the dry weight, and serves as a foundational component in staple food products [41]. In this study, the winter wheat starch content remained statistically invariant across all fertilization treatments—including sole nitrogen application (N) and combinations of N with mineral and manure fertilizers (NP, NPK, NM, M). This stability is consistent with Kaufman et al. [42], who reported starch concentrations of 75.3%, 74.2%, 74.7%, and 73.9% under nitrogen application rates of 0, 33, 66, and 100 kg N ha^−1^, respectively, with no significant differences among rates (*p* > 0.05).

Wheat grain protein content is a primary determinant of end-use quality—particularly baking performance—and nutritional value [43]. Sedimentation volume serves as a well-validated functional index of wheat quality, directly reflecting both the quantity and functional quality of gluten proteins (e.g., gliadin and glutenin ratios) [44]. In this study, the grain protein concentration was significantly elevated under sole N application relative to the control; values under the NM, NP, and NPK treatments were statistically comparable to those under N. Environmental factors—including temperature, solar radiation, and water availability—exert substantial influence on grain protein accumulation [45,46]. Consistent with Langenkämper et al. [47], the fertilizer regime significantly affected both grain protein and crude fiber content, with distinct responses observed between organic and conventional systems. Triboi et al. [44] further demonstrated that nitrogen fertilization enhances not only total protein but also the functional quality of storage proteins—specifically increasing the proportion and polymerization state of high-molecular-weight glutenins—thereby improving dough strength. Notably, the NM treatment yielded the highest sedimentation volume, followed sequentially by N, NPK, NP, and M—all significantly exceeding the control (*p* < 0.05). This pattern aligns with Lin et al. [48], who reported that long-term integrated organic–inorganic fertilization markedly enhanced both grain protein concentration and sedimentation volume in wheat. As Laidig et al. [49] emphasized, while genotype is the dominant factor governing sedimentation potential, fertilizer management constitutes a key modifiable agronomic lever for optimizing protein quality—particularly under uniform genetic backgrounds.

The gluten index is a standardized rheological parameter widely employed to assess the viscoelastic properties and functional quality of wheat gluten—particularly its resistance to deformation during dough mixing and fermentation. A higher gluten index generally reflects greater gluten strength, improved dough stability, and enhanced suitability for bread-making applications [50]. It is strongly correlated with key protein quality traits, including the proportion and polymerization state of high-molecular-weight glutenin subunits, and thus serves as a robust proxy for overall gluten functionality. In this study, the gluten index was significantly elevated under the NM, M, NPK, and NP treatments relative to CK, whereas no significant difference was observed between N and CK. While Ames et al. [51] established that the gluten index is predominantly genetically controlled and exhibits high heritability across environments, Vida et al. [52] demonstrated that nitrogen fertilization can significantly modulate gluten index expression—even in genotypes traditionally considered stable, such as winter durum wheat. Consistent with these findings, the present results confirm that both genotype and agronomic management—particularly nitrogen source and integration strategy—interactively govern gluten quality, with environmental conditions acting as an additional modulating factor.

Wet gluten refers to the viscoelastic protein fraction isolated from wheat flour through aqueous washing, representing the functional gluten network formed primarily by gliadin and glutenin polymers [53]. Its content is strongly correlated with the total grain protein concentration, though not strictly proportional due to compositional and structural variability in gluten proteins. In this study, the wet gluten content followed a pattern closely aligned with protein accumulation: it peaked under sole N application, followed sequentially by the NM, NP, and NPK treatments—all significantly exceeding CK (*p* < 0.05). Consistent with Lin et al. [48], integrated organic–inorganic fertilization (e.g., NM) and inorganic-only regimes (e.g., N, NP, NPK) both markedly enhanced wet gluten content relative to unfertilized CK. As nitrogen is a fundamental constituent of amino acids and thus indispensable for de novo protein synthesis, adequate N supply—particularly in readily plant-available forms—directly supports the biosynthesis and accumulation of gluten proteins. High-quality wheat grain—characterized by optimal protein quantity and functional quality—is critical for food processing and baking performance; it contributes not only to superior dough rheology and bread-making functionality but also to enhanced nutritional value, including improved dietary protein quality and bioavailability.

Figure 3 presents a trade-off analysis between the long-term mean maize yield and a composite grain quality index across the fertilization treatments. The integrated application of nitrogen and manure (NM) simultaneously enhanced both yield and quality—achieving the highest values for both metrics. In contrast, the NPK increased yield relative to the control but resulted in a comparatively lower grain quality. Treatments supplying nitrogen without balanced phosphorus and potassium—namely the N-only and NK—produced lower yields than NPK yet exhibited superior grain quality, exceeding even the NPK treatment in the quality index. The unfertilized CK yielded the lowest maize output and exhibited the poorest grain quality across all assessed parameters.

## 4. Materials and Methods

### 4.1. Experiment Regions

**Figure 4:**
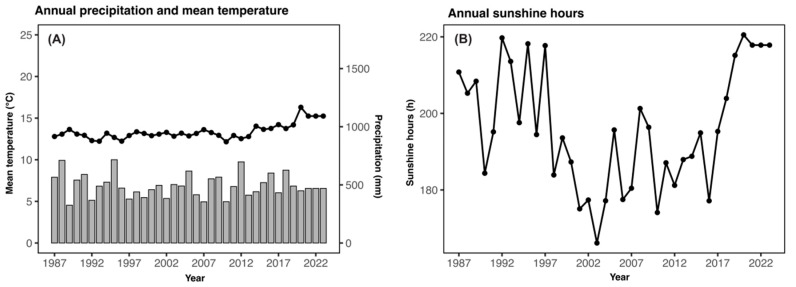
Long-term temporal dynamics of climatic variables in the sampling area from 1987 to 2023. (**A**) Changes in annual mean air temperature (°C) and total precipitation (mm). The black connected dots represent temperature, and the vertical gray bars represent precipitation. (**B**) Variations in annual sunshine hours (h).

The long-term field experiment was established in 1979 at the Wuqing Experimental Station of the Tianjin Academy of Agricultural Sciences (117.0° E, 39.2° N), located in the North China Plain. The site experiences a semi-humid, warm-temperate continental monsoon climate, characterized by a mean annual temperature of 11.6 °C, an annual sunshine duration of 2705 h, mean annual precipitation of 606.8 mm (with ~60% concentrated between May and September), and annual potential evapotranspiration of 1735.9 mm. The frost-free period averages 212 days. Comprehensive monthly meteorological records—including daily maximum, minimum, and mean air temperatures; 24 h accumulated precipitation; and sunshine duration—spanning the period 1978–2020 are presented in Figure 4.

### 4.2. Experimental Design

The long-term field experiment employed a winter wheat–summer maize rotation system. Six fertilizer treatments were assigned in a randomized strip-plot design with four replicates: (1) an unfertilized control (CK); (2) nitrogen only (N); (3) nitrogen + phosphorus (NP); (4) nitrogen + phosphorus + potassium (NPK); (5) sole manure (M); and (6) integrated nitrogen plus high-rate manure (NM). Urea (N: 46%), calcium superphosphate (P_2_O_5_: 12%), and potassium chloride (K_2_O: 60%) were applied as the respective sources of N, P, and K. In the NM treatment, organic amendments were updated over time to align with evolving regional resource availability and regulatory standards: farmyard manure blended with local soil was applied from 1979 to 1998 (N: 1.86%; P_2_O_5_:1.32%; K_2_O:1.60%); chicken manure was used from 1998 to 2015 (N: 1.53%; P_2_O_5_: 2.04%; K_2_O: 2.08%); and certified commercial organic fertilizer was adopted after 2015 (N: 1.39%; P_2_O_5_: 1.26%; K_2_O: 1.60%). Comprehensive nutrient specifications—including annual application rates (kg ha^−1^), timing relative to sowing, and chemical composition—are detailed in Table 3. All agronomic practices—including tillage, sowing, pest and disease management, and harvest—were implemented in accordance with locally validated recommendations throughout the 45-year experimental period (1979–2023). The maize was used as the initial crop in this study. Winter wheat was sown in late September or early October; summer maize was sown in mid-June on average; supplemental irrigation was applied three times—at the jointing, heading, and grain-filling stages—with each event delivering approximately 90 mm of water. Maize received no supplemental irrigation except under officially defined severe drought conditions.

### 4.3. Winter Wheat and Maize Stability Analysis

Grain samples of winter wheat and summer maize were harvested from the experimental plots, air-dried under ambient field conditions to a constant moisture content, and then weighed to determine the grain yield on a dry-weight basis. Grain yield stability across treatments was quantified using the coefficient of variation (CV, %), calculated as the ratio of the standard deviation to the mean yield (×100) over the 45-year experimental period (1979–2023). As established in Xu et al. [54], this metric effectively captures interannual yield variability attributable to climatic fluctuations, crop rotation effects, and long-term fertilizer management—enabling robust comparison of treatment resilience. The CV was computed as follows:

$$CV=\frac{Y_{\mathrm{std}}}{Y_{m}}\times 100$$

where *Y_std_* is the standard deviation of the crop yield of a particular treatment during the forty-five years of the experiment, and *Y_m_* is the mean yield of the same treatment during the same period.

### 4.4. Analysis of Grain Quality

Wheat grain quality parameters—including protein concentration, wet gluten content, gluten index, sedimentation value, and starch content—were determined for the 2020 harvest. Protein concentration was quantified via Kjeldahl nitrogen analysis (KDY-9820, Beijing Tongrunyuan Electromechanical Technology Co., Ltd., Beijing, China) following [55]. Wet gluten content and the gluten index were measured using a Glutomatic 2200 system (Perten Instruments, Stockholm, Sweden) in accordance with [56] and [57], respectively. Starch content was determined by enzymatic hydrolysis [58] using a Buhler laboratory mill (Wuxi Buhler Machinery Manufacturing Co., Ltd., Wuxi, China), and the sedimentation value was assessed with a BAU-A sedimentation meter (China Agricultural University, Beijing, China) per [59].

### 4.5. Statistical Analyses

Data are presented as mean ± standard deviation. Associations among wheat and maize yields, soil properties, and grain quality were evaluated using one-way analysis of variance (ANOVA) at a significance level of α = 0.05, implemented in SPSS Statistics 21.0 (IBM Corp., Armonk, NY, USA). Graphical representation and descriptive statistics of yield and grain quality data were generated using OriginPro 2018 (64-bit; OriginLab Corporation, Northampton, MA, USA).

## 5. Conclusions

A 45-year long-term field experiment conducted in a maize–winter wheat rotation system demonstrated that the NM consistently achieved the highest yields for both crops, followed closely by the NPK treatment; in contrast, the sole N application resulted in the lowest yields across both species. Notably, manure-only (M) application markedly enhanced the yield stability of winter wheat, whereas NM application conferred superior stability in maize, indicating crop-specific resilience benefits from organic–inorganic integration. Starch content remained statistically invariant across all fertilizer treatments—including organic (M), inorganic (N, NP, NPK), and integrated (NM) regimes—whereas the grain protein concentration was significantly elevated under the N, NP, NM, and NPK treatments relative to the control, with no significant differences among these four high-performing treatments. Furthermore, the NM treatment yielded the greatest improvements in functional quality indices: the gluten index and sedimentation volume—both key determinants of dough strength and baking performance. Collectively, these findings indicate that combining mineral nitrogen with manure not only maximizes grain yield but also enhances interannual yield stability and improves multiple dimensions of grain quality. Consequently, evidence-based, balanced fertilization management represents a strategic pathway to simultaneously advance productivity, resilience, and nutritional–functional quality, thereby supporting the sustainable intensification of cereal systems toward high-quality food security.

## Figures and Tables

**Table 1:** Wheat and corn yield under different fertilization regimes from 1979 to 2023.

	Period	CK	N	NP	M	NM	NPK
wheat							
**1979–1998**	1979–1983	2205.6 ± 158.3 b	3912.2 ± 370.4 a	5092.0 ± 229.9 a	4734.8 ± 267.7 a	4852.0 ± 133.2 a	4926.6 ± 180.0 a
	1984–1988	1376.0 ± 127.5 b	2927.0 ± 466.5 ab	4549.2 ± 564.3 a	4783.2 ± 811.4 a	4603.4 ± 402.0 a	4753.2 ± 365.5 a
	1989–1993	1602.8 ± 68.9 b	2738.8 ± 281.2 bc	4961.2 ± 445.5 a	4401.6 ± 782.7 ac	5688.4 ± 428.6 a	5310.4 ± 407.0 a
	1994–1998	1388.6 ± 158.3 b	1075.8 ± 206.8 b	4602.6 ± 613.9 a	2025.4 ± 286.2 b	5294.4 ± 417.6 a	5153.8 ± 493.1 a
**1999–2015**	1999–2003	2769.8 ± 194.6 a	2401.2 ± 490.0 a	5092.8 ± 1039.9 a	4060.0 ± 535.6 a	5598.8 ± 915.7 a	5456.8 ± 939.3 a
	2004–2008	1037.2 ± 245.6 b	805.8 ± 195.6 b	4007.8 ± 594.6 ac	2561.6 ± 357.7 bc	4705.2 ± 390.7 a	4567.4 ± 579.1 a
	2009–2013	1060.0 ± 166.0 c	1047.0 ± 262.0 c	4633.8 ± 147.5 a	2875.0 ± 352.1 b	4479.8 ± 267.0 a	4406.2 ± 268.4 a
	2014–2015	1287.9 ± 20.4 f	1731.3 ± 290.0 e	5159.1 ± 325.3 b	3563.4 ± 560.9 c	5479.4 ± 46.3 a	5432.5 ± 188.1 ab
**2016–2023**	2016–2020	1085.6 ± 330.9 d	1735.3 ± 672.8 d	4933.4 ± 2291.3 ab	3060 ± 281.0 bc	5819.8 ± 2028.2 a	5028.2 ± 2472.9 ab
	2021–2023	1616.3 ± 163.7 c	1236.1 ± 708.5 c	5883.3 ± 1342.6 a	3956.9 ± 1659.5 b	6273.6 ± 1419.4 a	5868.1 ± 1467.7 a
maize							
**1979–1998**	1979–1983	2984.4 ± 487.5 c	5618.6 ± 656.0 ab	6479.8 ± 552.1 ab	4221.5 ± 565.3 bc	6511.8 ± 611.7 ab	7039.0 ± 503.7 a
	1984–1988	2310.0 ± 257.8 b	5649.4 ± 662.8 a	6019.8 ± 673.5 a	4805.8 ± 916.9 ab	6272.0 ± 713.1 a	6429.4 ± 582.6 a
	1989–1993	1785.0 ± 242.9 c	3877.0 ± 372.9 bc	6157.4 ± 408.0 ab	6463.8 ± 980.3 a	6591.2 ± 456.7 a	6627.4 ± 483.9 a
	1994–1998	1608.6 ± 413.9 d	2810.2 ± 549.3 cd	4577.0 ± 409.9 abc	3079.6 ± 296.0 bcd	5018.4 ± 267.5 ab	5255.0 ± 452.3 a
**1994–2015**	1999–2003	2406.2 ± 290.9 b	4842.2 ± 451.0 a	5969.8 ± 549.3 a	5561.8 ± 335.6 a	6271.0 ± 529.3 a	6168.5 ± 591.9 a
	2004–2008	2761.2 ± 415.7 c	3655.8 ± 533.6 bc	5511.6 ± 337.1 ab	5085.0 ± 802.1 ab	6676.2 ± 379.0 a	6290.0 ± 382.3 a
	2009–2013	3466.8 ± 607.1 b	5742.0 ± 742.4 ab	7895.6 ± 898.1 a	6369.6 ± 1013.5 ab	9426.0 ± 827.1 a	8616.8 ± 844.5 a
	2014–2015	2960.0 ± 91.3 e	5805.9 ± 94.1 d	9125.9 ± 544.7 b	7667.8 ± 1111.6 c	10,758.4 ± 189.7 a	10,864.4 ± 58.1 a
**2016–2023**	2016–2020	3018.4 ± 582.2 e	6579 ± 395.7 d	8249.7 ± 1648.4 bc	7539.6 ± 1447.3 cd	9539.3 ± 1213.1 a	9595.5 ± 1415.8 ab
	2021–2023	2795 ± 655.1 f	5524.3 ± 610.7 e	8938 ± 1044.2 bc	7359.0 ± 1078.4 d	10,099.7 ± 316.3 a	9228.3 ± 312.2 ab

Note: The yield data are divided into three phases based on the types of applied organic fertilizers: 1979 to 1998: Farmyard manure mixed with local soil, 1999 to 2015: Chicken manure application, 2016 to 2023: Application of certified commercial organic fertilizer. Data are presented as 5-year averages. Different letters at the same index represent significant difference under different treatments (*p* < 0.05).

**Table 2:** The content of starch and protein in winter wheat grain under different treatments in 2020.

Treatment	CK	N	NP	NPK	M	NM
Starch (g 100 g^−1^)	68.6 ± 2.8 a	68.5 ± 3.0 a	68.3 ± 2.9 a	71.6 ± 0.5 a	71.9 ± 5.0 a	68.6 ± 2.9 a
Protein (%)	11.3 ± 0.1 c	13.6 ± 0.1 a	12.7 ± 0.2 b	12.4 ± 0.2 b	11.0 ± 0.2 c	12.6 ± 0.5 b

Note: CK (no fertilization), N (nitrogen), NP (nitrogen and phosphorus), NPK (nitrogen, phosphorus, and potassium), M (manure), NM (nitrogen plus manure combination). Different lowercase letters in column are significant differences at *p* < 0.05.

**Table 3:** Nutrient application rates for the cropping system at the experiment sites in 1979–2023.

Treatment	Corn Wheat							
	Inorganic N (kg N ha^−1^)	P_2_O_5_ (kg P_2_O_5_ ha^−1^)	K_2_O (kg K_2_O ha^−1^)	Organic Manure (kg ha^−1^)	Inorganic N (kg N ha^−1^)	P_2_O_5_ (kg P_2_O_5_ ha^−1^)	K_2_O (kg K_2_O ha^−1^)	Organic Manure (kg ha^−1^)
CK	0	0	0	0	0	0	0	0
N	210	0	0	0	285	0	0	0
NP	210	0	0	0	285	142.5	0	0
M	0	0	0	0	0	0	0	28,515
NM	210	0	0	0	285	0	0	11,535
NPK	210	0	0	0	285	142.5	71.3	0

## Data Availability

The original contributions presented in the study are included in the article. Further inquiries can be directed to the corresponding authors.
